# Possible linkage between neuronal recruitment and flight distance in migratory birds

**DOI:** 10.1038/srep21983

**Published:** 2016-02-24

**Authors:** Shay Barkan, Uri Roll, Yoram Yom-Tov, Leonard I. Wassenaar, Anat Barnea

**Affiliations:** 1 Department of Zoology, Tel-Aviv University, Tel-Aviv 61391, Israel; 2 School of Geography & the Environment, University of Oxford, South Parks Road, Oxford, OX1 3QY, United Kingdom; 3 Department of Zoology, University of Oxford, South Parks Road, Oxford, OX1 3PS, United Kingdom; 4 Department of Nuclear Sciences and Applications, International Atomic Energy Agency (IAEA), Vienna, Austria; 5 Department of Natural and Life Sciences, The Open University of Israel, Ra’anana 43107, Israel

## Abstract

New neuronal recruitment in an adult animal’s brain is presumed to contribute to brain plasticity and increase the animal’s ability to contend with new and changing environments. During long-distance migration, birds migrating greater distances are exposed to more diverse spatial information. Thus, we hypothesized that greater migration distance in birds would correlate with the recruitment of new neurons into the brain regions involved with migratory navigation. We tested this hypothesis on two Palearctic migrants - reed warblers (*Acrocephalus scirpaceus*) and turtle doves (*Streptopelia turtur*), caught in Israel while returning from Africa in spring and summer. Birds were injected with a neuronal birth marker and later inspected for new neurons in brain regions known to play a role in navigation - the hippocampus and nidopallium caudolateral. We calculated the migration distance of each individual by matching feather isotopic values (*δ*^2^H and *δ*^13^C) to winter base-maps of these isotopes in Africa. Our findings suggest a positive correlation between migration distance and new neuronal recruitment in two brain regions - the hippocampus in reed warblers and nidopallium caudolateral in turtle doves. This multidisciplinary approach provides new insights into the ability of the avian brain to adapt to different migration challenges.

Exposure to new information in adult animals has been positively correlated with the magnitude of new neuronal recruitment in their brains^1^,^2^. This exposure, linked to new neuronal recruitment, could be manifested in many ways throughout an animal’s life history and the challenges it faces^3^,^4^,^5^. The recruitment of new neurons is considered to be promoted by an elevated need to acquire new information in relevant brain regions^6^. Such recruitment may serve this purpose by facilitating brain plasticity and has been suggested to play an important role in acquiring new memories in both invertebrates^7^,^8^ and vertebrates^1^,^9^. Birds have been used as model animals in studies linking new neuronal recruitment with various behaviors, such as social complexity^10^, food hoarding^4^,^11^, song learning^12^, the reproductive cycle^13^, and others.

Long-distance migration is highly demanding in many aspects of a bird’s life cycle. Beyond the immense physiological effort required in traveling large geographical distances, birds also have to avoid predation, minimize resource competition, overcome adverse weather, and accurately navigate and orient^14^. The mortality rate of young avian migrants is far higher than that of their adult counterparts which suggests that learned experience is of great importance for migratory birds^15^. Indeed, several studies have explored the importance of learning and experience for accurate navigation in migratory birds^16^,^17^. Learning could be key to improved navigation ability; identification of suitable stop-over sites en-route; avoidance of harsh weather or potential predators; and for outcompeting both fellow migrants and local birds. Such needs for increased learning ability during migration may be answered by a greater neuronal recruitment in the relevant brain regions of migratory birds. To date, only two studies of passerines^18^,^19^ have directly investigated the possible relationship between neuronal recruitment and migratory behavior in birds. Those studies revealed that, for passerines, migrant species or sub-species possessed more new neurons than resident species in the brain regions that play a role in spatial information processing.

During the past century, great advances have been made in tracking and monitoring migratory birds. In addition to the increased use of recovery-based ringing and tagging, researchers nowadays have access to radio transmitters and satellite or GPS tracking to monitor individual birds^20^. Recently, the use of stable isotopes has been shown to be effective in quantifying individual or population migratory connectivity^21^,^22^. The use of stable isotopes to track migratory movement is based on the predictable global spatial patterns for hydrogen (H) and carbon (C) isotopes. Wherever a bird grows its feathers (often at its natal or overwintering site), the local isotopic patterns are translated through diet along the trophic cascade, and are fixed into the growing feathers, thereby reflecting the region the feathers were grown^23^. When a bird is caught later, its feathers’ ^2^H/H and ^13^C/^12^C ratios can be non-lethally analyzed and compared to known H and C isotopic distribution base-maps (e.g. isoscapes). This tissue-to-isoscape comparison is used to obtain estimates of the region where a bird had molted or grown its feathers. One of the primary advantages of the isotope technique is that every bird captured provides intrinsic spatial information about its migration route, without the need for the mark-recapture used in ringing and marking techniques^24^.

In this study we hypothesized a possible link between an increase in new neuronal recruitment and the distances traveled by migrant birds. We explored this phenomenon in reed warblers (*Acrocephalus scirpaceus*) and turtle doves (*Streptopelia turtur*), which are summer visitors in Israel and winter and molt in Africa^25^. Migration distance estimates were determined using feather isotopic ratios as an indicator of molt locality in the wintering grounds. New neuronal recruitment was evaluated in two brain regions known to take part in navigation and spatial orientation tasks-the Hippocampal complex (HC)^26^,^27^, and the nidopallium caudolateral (NCL)^27^.

## Results

### Migration distance and recruitment of new neurons

**Figure 1:**
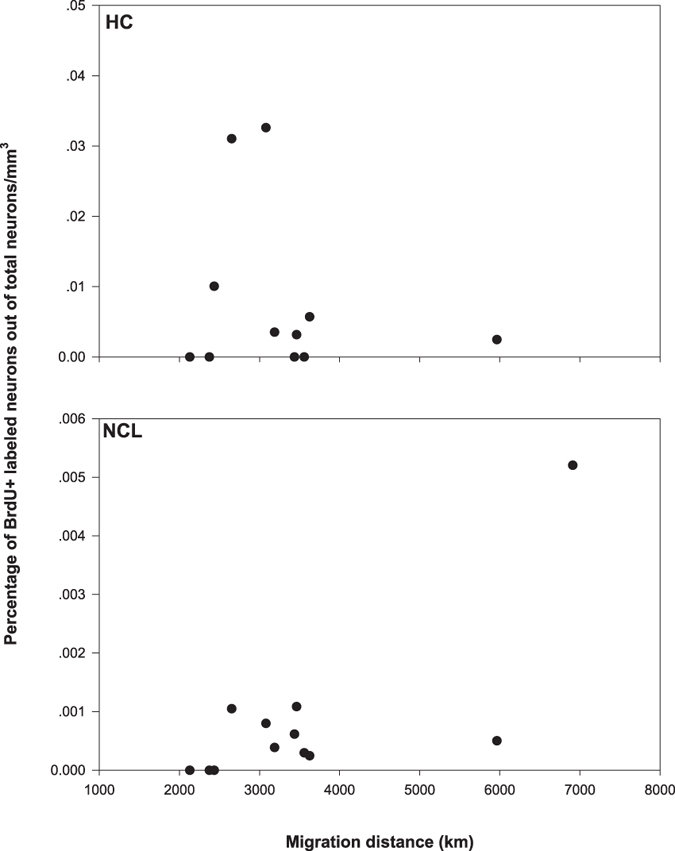
Percentage of new neuronal recruitment into the hippocampus (HC) and nidopallium caudolateral (NCL) of the turtle dove (*Streptopelia turtur*), as a function of the migration distances from their wintering grounds in Africa to Israel. For the HC, Spearman’s rank correlation, P = 0.96; *ρ* = −0.14 (N = 11) and for NCL P = 0.07; *ρ* = 0.53 (N = 12).

**Figure 2:**
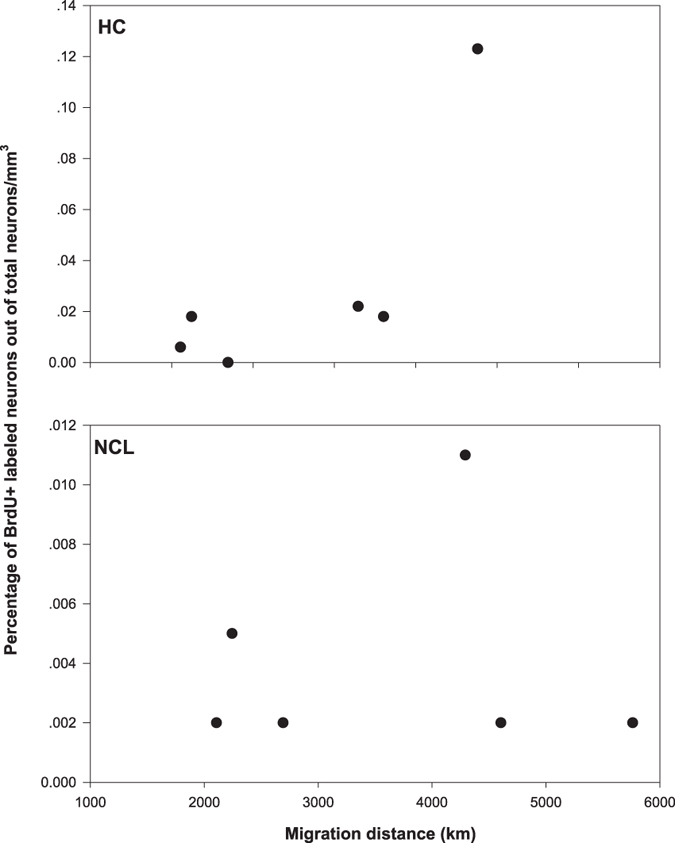
Percentage of new neuronal recruitment into the hippocampus (HC) and nidopallium caudolateral (NCL) of reed warbler (*Acrocephalus scirpaceus*), as a function of their migration distances from wintering grounds in Africa to Israel. For the HC, Spearman’s rank correlation, P = 0.12; *ρ* = 0.69 (N = 6), and for NCL P = 0.8; *ρ* = −0.13 (N = 6).

When exploring links between migration distance and new neuronal recruitment in turtle doves we found marginally significant increase in recruitment into the NCL for birds flying longer distances (P = 0.07; *ρ* = 0.53; N = 12; Fig. 1), but not in the HC (P = 0.96; *ρ* = −0.01; N = 11). In the reed warbler we found a similar but opposite trend, with increased neuronal recruitment into the HC (P = 0.12; *ρ* = 0.69; N = 6; Fig. 2), but not into the NCL (P = 0.8; *ρ* = −0.13; N = 6) of birds flying longer distances. Notably, these marginally significant results, respectively for the turtle doves and the reed warblers, were based on small sample sizes. For the turtle doves the statistical power for the NCL analysis was 76% at the nominal α  = 0.05 and 84.7% for α = 0.1. For the reed warbler analysis of the HC, the statistical power was low (35%), which meant that for our sample size (six birds), at the nominal rejection rate of 0.05 and the effect size we found, it was unlikely that we would obtain significant results. Upon setting our rejection rate to 0.1, the power of the test increased to 47.4%. In order to obtain a power value of 80% with the same effect size we would have needed about 10 birds in our sample at the 0.1 rejection rate or 12 birds for the 0.05 rejection rate.

### Wintering molt location

**Figure 3:**
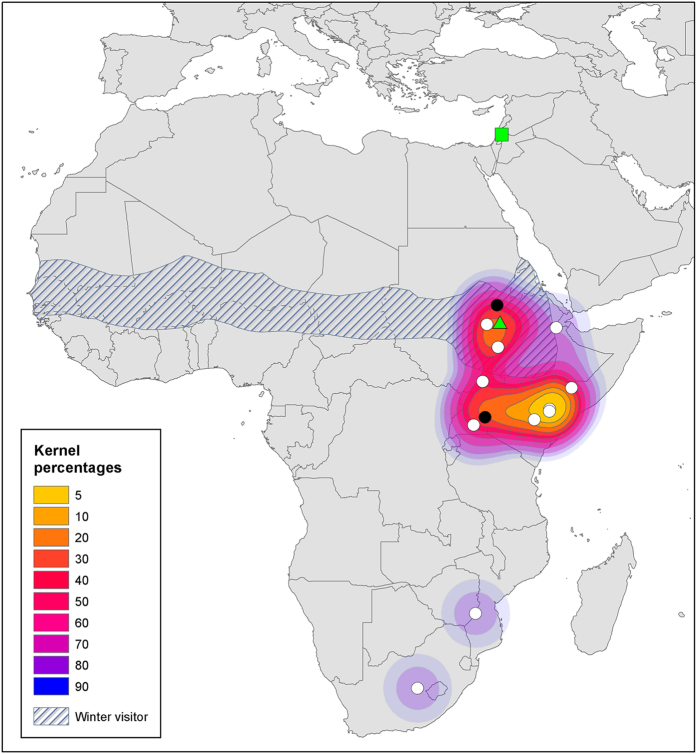
Wintering grounds of turtle doves (*Streptopelia turtur*) in Africa. Kernel percentages display probable wintering grounds based on C and H isotopic values derived from birds caught in Israel. Each dot represents the probable center of a single individual’s wintering range. Black dots represent birds that only provided isotopic information, white dots represent birds that also provided neuronal information. The striped area shows wintering grounds based on Urban *et al*.^28^ (Urban E.K., Fry C.H., Stuart, K. (1986). The birds of Africa. © A&C Black Publishers, used by permission of Bloomsbury Publishing Plc). The green triangle represents a single record of an individual that was ringed in Israel and caught in Africa. The green square represents the location in Israel where the birds were caught. Spatial analysis and map production were conducted in ArcGIS 10.1^56^.

**Figure 4:**
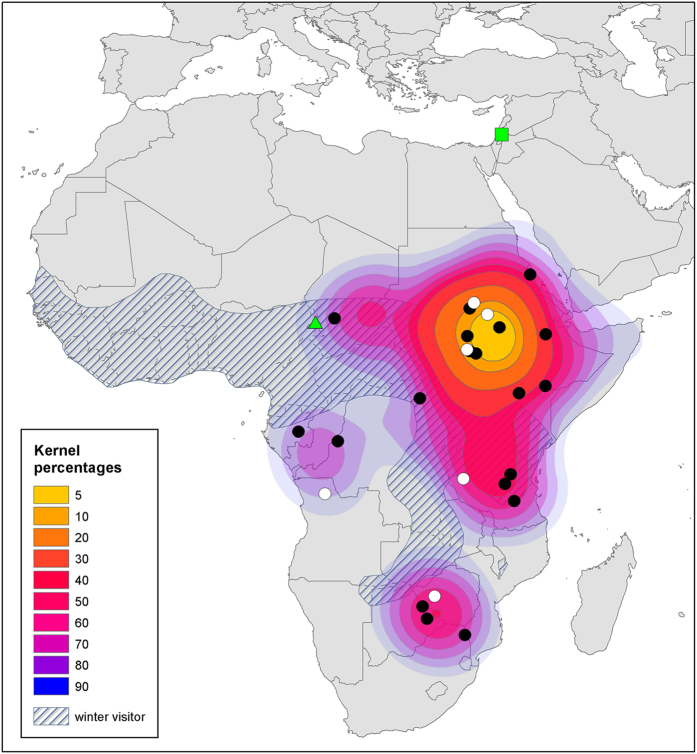
Wintering grounds of reed warblers (*Acrocephalus scirpaceus*) in Africa. Kernel percentages display probable wintering grounds based on isotopic values derived from birds caught in Israel. Each dot represents the probable center of a single individual’s wintering range. Black dots represent birds that only provided isotopic information, white dots represent birds that also provided neuronal information. The striped area shows wintering grounds based on Urban *et al*.^29^ (Urban E.K., Fry C.H., Stuart, K. (1986) The birds of Africa. © A&C Black Publishers, used by permission of Bloomsbury Publishing Plc). The green triangle represents a single record of an individual that was ringed in Israel and caught in Africa. The green square represents the location in Israel where the birds were caught. Spatial analysis and map production were conducted in ArcGIS 10.1^56^.

Only a few of the individuals that were analyzed for stable isotopes also underwent neuronal recruitment analysis (see below). We used our larger sample size of individuals whose feathers were measured for stable isotopes (25 reed warblers, 14 turtle doves), to estimate probable wintering grounds in Africa for these two species. Figs 3 and 4 display kernel distributions of wintering grounds for reed warblers and turtle doves, respectively. The maps also show data from ringing returns (obtained from the Society for the Protection of Nature in Israel – www.natureisrael.org), as well as known wintering grounds based on expert drawn maps^28^,^29^.

## Discussion

In this work we found a tentative link between the migration distance of two bird species and new neuronal requirement into the brain regions that play a role in spatial orientation and navigation. While our sample sizes are inevitably small (see also below), we found in two distinct regions – the HC for reed warblers and the NCL in the turtle doves, an increase in neuronal recruitment corresponding with increased migration distance. Our combined approach, though innovative, employed two commonly used and accepted isotope and neuronal analyses methods^19^,^30^.

As noted above, the limitation of our results is that they are based on small sample sizes. Furthermore, just a few data points were responsible for much of the observed effect, both in the HC of reed warblers and the NCL of turtle doves (Figs 1 and 2). However, these data points are not the outcome of any histological failure, because our brains were processed simultaneously in few batches, and these data points were in batches that included other brains, which yielded data points with different scores. Moreover, statistically, these data points did not depart from the central tendency of the other values and were not designated as an outlier when fitted to a Poisson distribution^31^. Therefore, despite the small sample sizes and the consequent low statistical power obtained, we suggest that our data might indicate a general phenomenon. This suggestion is manifested in the fairly high coefficients of determination – *ρ* (0.53 for the turtle doves in the NCL and 0.69 for the reed warblers in the HC), for the two separate tests. Nevertheless, further investigation using larger datasets in different regions and organisms will be critical to obtain additional support for our hypothesis.

While a larger sample size would have been desirable in order to confirm our hypothesis, the experimental design and field logistics made this impossible. Initially, we sampled only adult birds caught in the wild within a particular season. Furthermore, these birds had to survive 35 days of captivity, during which new neurons migrated and were incorporated into the designated brain regions.

The analysis we conducted targeted neuronal differences within populations of the same species. Such differences probably arose due to evolutionary adaptations to different migration distances. As such, this phenomenon somewhat differs from cases in which new neuronal recruitment is temporally tuned to answer a specific need^10^,^13^,^32^. Our results indicated a variance in the degree of plasticity in the brain, potentially derived from genetic differences between populations migrating longer or shorter distances. A similar mechanism of differences in new neuronal recruitment between populations of the same species has been recorded with respect to food hoarding in black-capped chickadees (*Poecile atricapilla*) experiencing different climatic conditions^33^,^34^.

The HC has been shown in previous works on passerines to be important for the processing of long-term memory, navigation, and spatial orientation^1^,^26^. Similarly, the NCL has been found to be involved in processing spatial information^27^,^35^ and working memory^36^,^37^ in pigeons. Thus, our findings that the HC and the NCL are important for navigation and orientation in reed warblers and turtle doves, respectively, are in line with previous results. However, to the best of our knowledge, passerine NCL has never been tested with respect to orientation and navigation. Our results show that, at least for reed warblers, the NCL plays a minor role in these brain functions relative to the HC. Doves were previously shown to depend on the HC for navigation across a familiar space within 10–20 km from the home loft, whereas long-distance navigation over unfamiliar spaces was HC independent^26^. Therefore, turtle dove migration between Africa and Israel is unlikely to be HC-dependent, and indeed we did not find any link between neuronal recruitment in the HC and migration distances traveled by this species (Fig. 1).

The differences in the brain regions found to be significantly correlated with migration distance in the two species might be explained by differences in their migratory behavior. Reed warblers are lone nocturnal migrants that rely on self-orientation for navigation. Conversely, turtle doves tend to migrate in large flocks, mostly at night but also during the day^38^. The evolutionary advantage of flocking lies in superior group decisions, known as the “many wrong” principle, in which pooling information from many inaccurate compasses yields a single more accurate compass^39^. In addition, group flight may also assist in locating limited or obscured landmarks while flying^40^, and allow inexperienced individuals to follow experienced partners^41^,^42^. Such information sharing between flock members may reduce the navigational investment per individual, but requires developed social interaction and communication among migrating individuals^40^,^43^. The NCL has been suggested to take part in the integration of visual and auditory pathways^44^,^45^ and may play a primary role in social interactions. Flock size is a plastic social structure which has been found to positively correlate with migration distance in many avian species^46^,^47^. Flocking migrants that fly longer distances could thus need to acquire more information on more complex social structures. This may explain the link we found in doves between neuronal recruitment in the NCL and migration distances. Beyond their behavioral differences, turtle doves and reed warblers belong to clades that are thought to have been separated from one another other ~85 million years ago^48^, that may have resulted in a parallel evolution manifested in similar functions of different brain regions.

Using information from two stable isotopes, as well as spatial averaging via use of kernel estimations, we were able to ascribe probable localities of the winter molt for each individual bird. Furthermore, our analysis and methodology enabled us to determine the wintering grounds of the populations of the two species under study - the reed warbler and the turtle dove. Our findings add information about the geographical extent of these species during winter in a region that is lacking in research and surveys. Only 60 returns for ringed reed warblers and six returns for turtle doves from Europe and the Middle East have been recorded in Israel over the past 20 years, with two ringing returns for these two species from all of Africa (Figs 3 and 4). Moreover, the maps we constructed based on isotope analysis have proven similar to the limited published information^28^,^29^ and ringing data, thereby supporting our methodology. Wintering ground maps are particularly important for turtle doves as there is a growing concern regarding the global decline of populations of this species^49^. Identifying the wintering grounds of this species could aid in their future conservation measures.

Here we present, for the first time, a preliminary indication of a linkage between distances traveled and new neuronal recruitment between different populations of migrating birds. Our evidence suggests a possible important adaptation of migrating birds – those that travel greater distances exhibit greater brain plasticity. Obviously, more work is needed in different species, habitats, and brain regions, in order to substantiate our findings. Nonetheless, our methodologies and results can serve as a first important step. This work is also of value in light of the ongoing climatic, enviromental and landuse changes, as an indication that pre-adaptation towards greater brain plasticity could prove advantagous for species or populations in novel enviroments or ecosystems. We hope that our work will promote additional research on the effects of migration on the avian brain.

## Methods

### Experimental design

Our neuronal analysis was carried out on six adult reed warblers and twelve adult turtle doves, collected under the Israel Nature and National Parks Protection Authority permit (2005/24706). The study was approved by the Tel Aviv University Institutional Animal Care and Use Committee (permit L-06-008) and was performed in accordance with its regulations and guidelines regarding the care and use of animals for experimental procedures. Birds were caught with mist nets in the Jordan Rift Valley, Israel (32°.41′N; 35°.53′ E) during spring (February to April) and summer (June to July) between the years 2005–2009. Birds were aged according to plumage, iris, and leg color^25^,^50^. We used only adult birds in order to avoid possible effects of age on new neuronal recruitment and to ensure that the sampled individuals had molted their feathers at least once in Africa (see below). Two tail feathers were collected from each individual and tested for stable isotopes (Carbon: *δ*^13^C and Deuterium: *δ*^2^Η). Isotopic data were compared to the respective isoscapes of Africa (see below). Brains of all birds were processed and analyzed for new neuronal recruitment.

### Stable isotope analysis

Two tail feathers were sampled from each individual for the analysis of stable isotope remains (*δ*^2^H; *δ*^13^C). Feather H isotopic composition for non-exchangeable H was analyzed following the comparative equilibration method described in Wassenaar and Hobson^51^. All feathers were cleaned of surface oils using a 2:1 chloroform:methanol solution and then dried overnight in a hood. For the *δ*^13^C analysis, we used 1.2 ± 0.2 mg subsamples of the feathers (weighed by microbalance - Sartorius SE2; Gottingen, Germany) and placed in tin capsules (D1007; Elemental microanalysis; UK). For the *δ*^2^H analyses, subsamples weighing 0.35 ± 0.01 mg were placed in silver capsules (D1007; Elemental microanalysis; UK). Samples were then analyzed on a Europa 20:20 continuous-flow isotope-ratio mass spectrometer (CF-IRMS) interfaced with a Robo Prep elemental analyzer. *δ*^13^ C measurements were reported in *δ*-notation relative to the Pee Dee Belemnite standard (PDB) in parts per mil deviations (‰). Measurement error is estimated at 0.1‰ and 0.3‰ for the *δ*^13^C values. The *δ*^2^H measurements were reported as parts per mil deviations (‰) relative to Vienna Standard Mean Ocean Water–Standard Light Antarctic precipitation scale (VSMOW-SLAP). The H isotope analysis is more complex than that of *δ*^13^C due to the problem of uncontrolled isotopic exchange between feathers and ambient water vapor^51^. To correct this effect, accepted keratin standards were used so that the *δ*^2^H values reported here correspond to non-exchangeable feather hydrogen. This gave us *δ*^13^C and *δ*^2^H values for each individual, of the two species. Reed warblers and turtle doves are known to molt during winter in Africa^52^,^53^, and we therefore allocated their feathers’ isotope values to this continent. Feather isotope values were matched to precipitation isotope values, and a constant isotope fractionation factor of +25‰ was added for *δ*_2_H feathers values^51^ and +1‰ for *δ*^13^C^54^.

### Spatial analysis

To define the wintering molting regions for all birds we used ‘map lookup’^55^ and spatial averaging approaches. This entailed comparing the two isotopic values measured in the feathers of each bird to their parallel values in the known isoscapes, in order to create a probable molt region for each isotope. Regional *δ*^2^H isoscapes for the relevant feathers molting months (October–December) were obtained from WaterIsotopes.org web page, while *δ*^13^C annual isoscape was provided by Dr. C. Still (Department of Geography & Institute for Computational Earth System Science UC Santa Barbara, USA).

Initially, we extracted for each bird and each of the two isotopes studied, those values of the isoscapes that corresponded to the measured values in the feathers up to the precision level of the isoscape layer. Thus, if our measured value was X we extracted from the isoscape layer only those values that X lay between – i.e. those values immediately preceding or following X. All of these extracted values for each bird were spatially compared between the two isotopes studies. In cases where there was a distance of 10 km or less between these extractions for the two isotopes a possible location was indicated. The natural spatial spread of both ^2^H and ^13^C in Africa does not show a clear east-west gradient. Consequently, for some individuals our approach found several possible locations on both the eastern and western sides of the continent that corresponded to their feathers’ isotopic values. However, previous studies have shown that reed warblers and turtle doves migrating across the eastern Mediterranean sea (where the birds were caught), spend the winter in eastern Africa^28^,^29^. Thus we excluded from our analysis those possible locations which were west of the 18 ^o^E longitude. For each bird all possible locations eastern of 18 ^o^E longitude, were spatially averaged using a kernel density smoothing method. This method ultimately averages out those locations further away from the majority of all matches and produces a single potential point (as the centroid of the 10% kernel volume contour) which was then used as the location of the wintering ground for that bird. We next measured the distance between this point and the locality where the bird was captured in Israel (using an equal distance global projection) in order to determine the minimum migration distance traveled by each bird. We later used all the centroid locations of all the birds for each species to produce a map of the wintering grounds of the two species. All spatial analysis was conducted in ArcGIS 10.1^56^.

### Neuronal recruitment analysis

**Figure 5:**
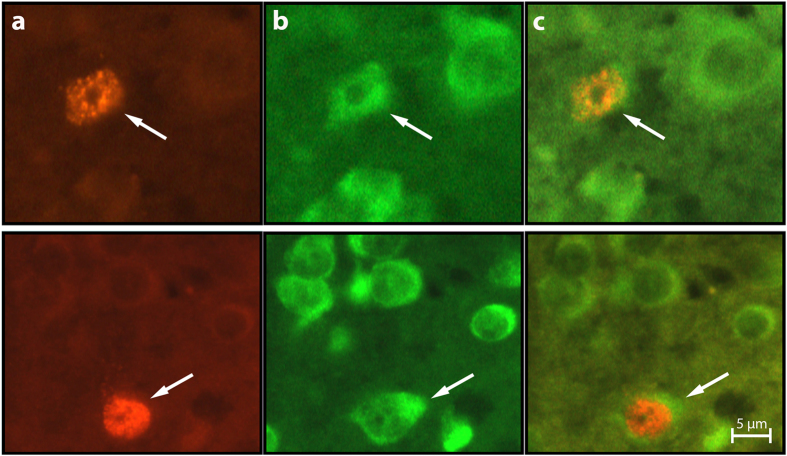
New neurons at three microphotographs of the same field, in the nidopallium caudolateral (NCL; upper row) of turtle dove (*Streptopelia turtur*) and the hippocampus (HC; lower row) of reed warbler (*Acrocephalus scirpaceus*). BrdU-labeled cells were identified with a rhodamine filter (**a**) and Hu-labeled neurons were identified with a FITC filter (**b**). Double-labeled neurons were identified by alternating between these two filters and by using a dual FITC-rhodamine filter to show co-localization of the two markers (**c**).

Following capture, the birds were transferred to outdoor aviaries in the Botanical Gardens of Tel Aviv University and injected three times, at 24 hours apart, with the cell birth-date marker 5-bromo-2-deoxyuridine (BrdU). Five weeks post-BrdU treatment birds were killed with an overdose of anesthesia and their brains underwent through histological procedures in which they were embedded in polyethylene glycol, blocked and cut transversely at thickness of 6 μm, along the rosto-caudal axis. Then, brain sections went through immunohistochemistry procedures that stained all neurons with fluorescent green (with anti-HuC/HuD), and nuclei of new neurons with fluorescent red (with anti-BrdU). Therefore, cells with co-localization of green cytoplasm and a red fluorescent nucleus were identified as new neurons (Fig. 5). For full details of the histological and immunohistochemical protocols used for both species, see Barkan *et al*.^19^.

### Brain mapping and quantification

**Figure 6:**
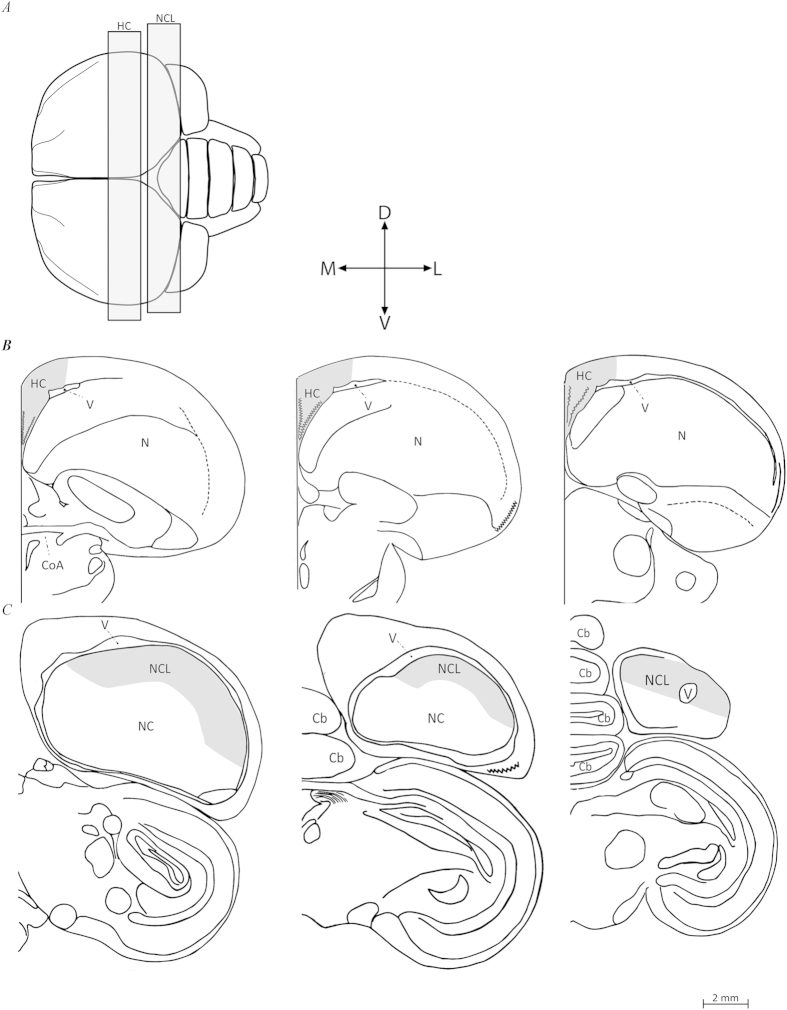
Schematic views of the two investigated brain regions in brains of turtle doves (*Streptopelia turtur*). (**A**) Top view of the brain: rostral is to the left, caudal is to the right. We indicate the range within which frontal sections were taken from the hippocampal complex (HC), and nidopallium caudolateral (NCL). Seven sections were sampled along the rostro-caudal axis of each brain region (for details, see text), three of which are shown here: the most rostral, the middle, and the most caudal (from left to right), in HC (**B**), and NCL (**C**). Abbreviations: Cerebellum (Cb), Commissura anterior (CoA), Nidopallium (N), Nidopallium caudale (NC), Lateral ventricle (V). Orientations: Dorsal (D), Lateral (L), Ventral (V), and Medial (M). Created from images originally appearing in: Karten, Harvey J., and William Hodos^57^. *A Stereotaxic Atlas of the Brain of the Pigeon (Columbia Livia)*. pp. 47-54, 61-67. © 1967 The Johns Hopkins Press. Adapted and reprinted with permission of Johns Hopkins University Press.

In both species, the brain regions we examined were the HC and NCL. In turtle doves, for each brain region, we defined and examined the most rostral and caudal section, and examined five additional sections between them, separated by an average distance of 240 μm (Fig. 6). For the HC, the most rostral section was defined by the presence of the commissura anterior (CoA), and corresponded to level A7.75 in the atlas of the pigeon brain^57^, and the most caudal section of the HC corresponded to level A6.25 in that atlas (Fig. 6B). The ventral, dorsal, and medial boundaries in each section were defined according to previously defined criteria^4^. For the NCL, the most caudal section corresponded to level A3.0 in the atlas of the pigeon brain^57^. The most rostral section corresponded to level A4.5 in this atlas, and was defined according to the lateral ventricle, along the dorsal part of the brain (Fig. 6C). The ventral boundary was indistinguishable by our staining methods and was therefore determined according to the dopaminergic innervation recorded in pigeons^58^. Since brains of laughing doves and turtle doves differ in size from the pigeon brain, we followed the method we previously used^19^ to determine the ventral boundary relative to that in the pigeon brain. For the definition of HC and NCL in reed warblers, a similar procedure was performed, as described in our previous publication^19^.

In both species, we used a computerized brain-mapping system (Stereo Investigator; MicroBrightField Inc.) to draw the boundaries of the HC and NCL in each section sampled, mark the position the new neurons and quantify their number. Total neuronal density was quantified in one section in each brain region, by counting all neurons within 12–18 sampling squares (100 × 100 μm each), randomly chosen by the software, using the fractionator probe. Our measure of new neuronal recruitment was calculated as a percentage of new out of total neurons per mm^3^, in these two brain regions.

### Statistical analysis

Non-parametric Spearman’s rank correlation was conducted between values of new neuronal recruitment and the calculated distances the birds had travelled. These regressions were conducted separately for the two species and each of the brain regions examined. We used the forward discovery rate correction for multiple testing^59^ to assign significance, using α = 0.05 as our target for rejection of the null. We then conducted power analyses on these four separate tests. All statistical analyses were conducted in R^60^.

## Additional Information

**How to cite this article**: Barkan, S. *et al*. Possible linkage between neuronal recruitment and flight distance in migratory birds. *Sci. Rep*. **6**, 21983; doi: 10.1038/srep21983 (2016).
